# In depth characterization of midbrain organoids derived from wild type iPSC lines

**DOI:** 10.1371/journal.pone.0292926

**Published:** 2023-10-20

**Authors:** Ivan Pavlinov, Mitali Tambe, Joshua Abbott, Ha Nam Nguyen, Miao Xu, Manisha Pradhan, Atena Farkhondeh, Wei Zheng

**Affiliations:** 1 National Center for Advancing Translational Sciences, National Institutes of Health, Bethesda, MD, United States of America; 2 Institute for Cell Engineering, Johns Hopkins University School of Medicine, Baltimore, MD, United States of America; 3 3Dnamics, Inc., Baltimore, MD, United States of America; Affiliated Hospital of Jiangsu University, CHINA

## Abstract

The ability to model human neurological tissues in vitro has been a major hurdle to effective drug development for neurological disorders. iPSC-derived brain organoids have emerged as a compelling solution to this problem as they have the potential to relevantly model the protein expression pattern and physiology of specific brain regions. Although many protocols now exist for the production of brain organoids, few attempts have been made to do an in-depth kinetic evaluation of expression of mature regiospecific markers of brain organoids. To address this, we differentiated midbrain-specific brain organoids from iPSC-lines derived from three apparently healthy individuals using a matrix-free, bioreactor method. We monitored the expression of midbrain-specific neuronal markers from 7 to 90-days using immunofluorescence and immunohistology. The organoids were further characterized using electron microscopy and RNA-seq. In addition to serving as a potential benchmark for the future evaluation of other differentiation protocols, the markers observed in this study can be useful as control parameters to identify and evaluate the disease phenotypes in midbrain organoid derived from patient iPSC-lines with genetic neurological disorders.

## Introduction

One of the critical limitations to drug discovery for neurological diseases is the absence of biologically relevant disease models [1]. This is especially true for genetic disorders which have historically been modeled using genetically altered animals or human immortalized lines which often do not accurately recapitulate disease phenotypes [2–4]. Patient hiPSC-derived 3D tissue-like aggregates, or organoids, can overcome some of these hurdles by recreating the architecture and function of the human brain as well as capturing the genetic factors that impact tissue differentiation and lead to a diseased phenotype [5–8]. Many different tissue types have been modeled using organoids, and methods for generating more complex or regiospecific organoid types continue to expand [9].

Although brain organoids can more closely mirror the human brain than alternative models, challenges remain in creating truly brain-like tissues reproducibly. Brain organoids are initially generated from embryoid bodies and thus contain a variety of precursor cells making it difficult to generate region specific organoids [10]. The organoids also contain a mixture of cells at different developmental states, limiting their ability to recapitulate mature brain function [11, 12]. Further, while more complex than a 2D cellular model, the 3D structure of the cells in organoids may not allow for biologically relevant cell connectivity and communication [13, 14]. Taken together these factors make it difficult to grow a large number of reproducible organoids across genetically different hiPSC lines for their use in disease modeling and preclinical drug development. Additionally, quality control criteria need to be established to determine whether a particular generation protocol can yield high quality region-specific brain organoids.

Several different methods have been developed to try to overcome this limitation [15–17], including methods that use pre-differentiated NPCs [18, 19]. Recently, a protocol using a miniaturized bioreactor was developed to generate region-specific brain organoids [20]. This matrix-free method reduced the necessary culture volume, increased throughput and reproducibility of generation which has enabled the use of these region-specific brain organoids for drug discovery [21, 22]. In this paper, we aim to broadly evaluate this protocol’s ability to generated midbrain organoids from three genetically different iPSC lines over a 90-day period using immunohistochemistry, immunofluorescence, electron microscopy, and RNA-seq. We demonstrate that the organoids express mature regiospecific biomarkers such as GFAP, TH and GABA as well as presence of similar extra- and intracellular features of primary neuronal tissues. Additionally, we show that the protocol reproducibly generates organoids that are transcriptionally similar to each other when they are generated from the same hiPSC line while still being distinguishable from organoids generated from other lines. These results can be used as a benchmark for the evaluation of midbrain organoids generated with different protocols and should further enable the use of midbrain organoids as a disease model system for drug efficacy evaluation.

## Materials and methods

### Cell lines and culture

Dermal fibroblasts were obtained from Coriell Cell Repositories (GM05659) and AATC (CRL-2708, PCS-201-012) and cultured in fibroblast media (DMEM, 10% fetal bovine serum, 100 units/mL penicillin and 100 μg/mL streptomycin) in a humidified incubator with 5% CO_2_ at 37°C. Fibroblasts were reprogrammed into iPSCs using the non-integrating Sendai virus technology [23]. Human iPSCs were cultured in mTeSR™1 (STEMCELL Technologies) on Matrigel (Corning, 354277)-coated plates at 37°C in humidified air with 5% CO2 and 5% O2.

### Midbrain generation from iPSC

Midbrain organoids (MOs) were generated from iPSCs using a published method [20]. Briefly iPSCs were dissociated into single cells with Gentle Cell Dissociation Reagent (GCDR; Cat# 07174, STEMCELL, 100ml/bottle)and plated into an Aggrewell plate (Stemcell Technologies Inc.) at 3x10^6^ cells per well. The plate was spun down according to manufacturer’s recommendation in containing midbrain differentiation medium #1 supplemented with 5 μM ROCK inhibitor Y-27632 (Tocris 1254). Spheroids were collected and transferred into a ultralow attachment 6-well plate (Corning) containing midbrain differentiation medium #1 and placed on an orbital shaker rotating at 120 RPM in an incubator. After 5 days, medium was replaced with midbrain differentiation medium #2. Next, the plate was placed on an orbital shaker rotating at 120 rpm in an incubator. On day 7, the media was replaced with midbrain differentiation medium #3. Generation of midbrain organoids was repeated three separate times and samples were pooled for all downstream processing except for RNAseq.

### Embedding, sectioning and IHC

Embedding, sectioning and IHC were performed using a previously published protocol [24]. MOs were harvested at specific timepoints and fixed in 4% PFA. MOs were incubated overnight in 20% glycerol and 2% DMSO to prevent freeze-artifacts. The specimens were then embedded in a gelatin matrix using MultiBrain®/ MultiCord® Technology (NeuroScience Associates, Knoxville, TN). Blocks were rapidly frozen, cured by immersion in 2-Methylbutane, chilled with crushed dry ice, and mounted on a freezing stage of an AO 860 sliding microtome. The MultiBrain®/ MultiCord® blocks were sectioned in coronally on the microtome. All sections were cut through the entire length of the specimen segment and stored in Antigen Preserve solution (50% PBS pH7.0, 50% Ethylene Glycol, 1% Polyvinyl Pyrrolidone) until staining.

For IHC, the sections were immunostained with primary antibodies (Table 1) in staining buffer (1x TBS (Invitrogen with 0.3% Triton X-100 (Sigma Aldrich)) overnight at room temperature. Sections were washed thrice in 1x TBS and incubated with a fluoro-tagged or biotinylated secondary antibody (Table 1). Sections stained with a biotinylated secondary antibody were treated with a fluorescent tagged streptavidin and washed thrice with 1x TBS. All sections were then mounted on gelatin coated glass slides and air dried. Slides were dehydrated in alcohols, cleared in xylene, coverslipped and imaged

**Table 1:** Table of antibodies used for IHC and IF.

Antibody	Host	Source	Catalog	Concentration
Anti-tyrosine hydroxylase	Rabbit	Pel Freez	P40101	1:18000 (IHC), 1:500 (IF)
Anti-FOXA2	Goat	R&D Systems	AF2400	1:1500 (IHC), 1:500 (IF)
Anti-caspase-3	Rabbit	Cell Signaling	9661	1:250 (IHC) 1:1500 (IF)
Anti-βIII tubulin (TUJ1)	Chicken	Abcam	ab41489	1:500 (IF)
Anti-GABA	Rabbit	Sigma Aldrich	A2052	1:100000 (IHC), 1:50000 (IF)
Anti-GFAP	Chicken	EnCor	CPCA-GFAP	1:150000 (IHC), 1:5000 (IF)
μ-Opioid Receptor IHC	Rabbit	Abcam	ab140911	1:10000 (IF)
Anti-rabbit biotinylated	Goat	Vector	BA-1000	1:1000 (IHC)
Anti-goat biotinylated	Rabbit	Vector	BA-5000	1:1000 (IHC)
Anti-chicken biotinylated	Donkey	Jackson Labs	703-065-155	1:1000 (IHC)
Anti-rabbit AlexaFluor 555	Donkey	ThermoScientific	A31572	1:500 (IF)
Anti-goat AlexaFluor 647	Donkey	ThermoScientific	A21447	1:500 (IF)
Anti-chicken AlexaFluor 647	Donkey	Jackson Labs	703-605-155	1:500 (IF)
Anti-chicken AlexaFluor 555	Donkey	Jackson Labs	703-165-155	1:500 (IF)

### Transmission electron microscopy

90-day MOs were prepared and imaged based on a previously published protocol [24]. MOs were fixed in 0.1 M Sodium Cacodylate buffer containing 2% glutaraldehyde and 4% formaldehyde for an hour at room temperature. Whole MOs were transferred to a glass vial and washed twice in 0.1M Sodium Cacodylate buffer for 10 minutes. Next, organoids were post-fixed in the dark with 1% Osmium Tetroxide for an hour and washed twcie as described previously. Next, MOs were washed once in 0.1N Sodium Acetate buffer and stained with 0.5% Uranyl Acetate for an hour in the dark. After *en block* staining, organoids were washed with 0.1N Sodium Acetate buffer twice for 10 min, followed by gradual dehydration. Briefly, MOs were washed twice for 10 mins each in 35%, 50%, 70% and 95% ethanol, followed by three final 10 min washes with 100% ethanol. MOs were further dehydrated with three 10 min washes in Propylene oxide, followed by overnight infiltration in 50:50 epoxy resin/ propylene oxide solution at room temperature.

The next day, each MO was removed from the infiltration solution, blotted, and embedded in a plastic mold containing 100% epoxy resin (PolyScience Resin). Then MOs, were transferred to a 55 ˚C oven and incubated for 48 hours. Next, each organoid embedded in resin mold was sectioned into 70 nm ultra-thin sections using the UC6 Leica Microtome. The sections were transferred to a 150-copper mesh grid and were examined under a Hitachi H7600 transmission electron microscope. The grids were post-stained with 1:1 0.5% Uranyl Acetate in ddH2O and 70% Ethanol for 2 min and then rinsed with ddH2O four times. Next, the organoids were stained with 50%Lead Citrate solution in ddH2O for 2 min and rinsed with ddH2O four times. The grids were carbon coated with a TedPella/Cressington Evaporator and imaged on a Hitachi H7600 transmission electron microscope at 80 KeV.

### Scanning electron microscopy

90-day MOs were fixed and dehydrated as described above. After the 100% Ethanol rinse, organoids were air-dried three times for 10 mins each with Tetramethylsilane under the hood. Next, each MO was carefully attached SEM stub usingcarbon tape and transferred to an Edwards vacuum system. Samples were then uniformly coated with highly conductive Au/Pd metal using a rotary tilting stage for 3 minutes. After Au/Pd coating, each organoid was transferred to a Hitachi S4500 scanning electron microscope for higher resolution imaging at 5.0 KeV.

### RNA extraction and RNA-seq

Total RNA was extracted from 90-day MOs using RNeasy Midi kit (cat #75144, Qiagen, Gaithersburg, MD). RNA quality was determined using a Bioanalyzer 6100 (Agilent, Santa Clara, CA). Directional RNA-seq was performed with 100 ng of total RNA using the TruSeq Stranded mRNA Library Prep Kit (Illumina, San Diego, CA), and 125 bp end sequence readers were generated using the HiSeq 2500 platform (Illumina, San Diego, CA).

### IHC image analysis and quantification

Image analyses were performed using the HALO software (IndicaLabs) as previously described [24]. Area Quantification module was used to quantify the positively stained region for each slide. Analysis parameters (signal intensity ranges for low, moderate, and weak intensities) were set and applied to all images for a given stain. The finalized analysis algorithm was run on all images and the generated data were exported and organized in a Microsoft Excel spreadsheet. All graphs and summary data were generated from the raw data using GraphPad Prism 9.

### RNA-seq analysis

RNA-seq analysis was performed as described previously [24]. FASTQ files were evaluated for QC using FastQC (v0.11.5), raw reads were then trimmed using theIllumina adapter in Trimmomatic (v0.36). Sequence alignment was performed using STAR (v2.5.2.a) with reference assembly GRCh38.p3 and Ensembl annotation 82 with ENCODE options.

All secondary analyses were performed using R (version 4.0.2). Genes were filtered to keep only genes with more than 1 count per million (CPM) in at least 75% of the samples. PCA was then performed using PCAtools (v2.4.0) on the TMM normalized countsusing edgeR (v3.16.5). Statistical analysis to determine differentially expressed genes was performed with limma (v3.48.1). Dispersion estimation was performed using the voom function and statistical analysis was completed using eBayes. Genes were considered differentially expressed if log2 fold change was > |1| with a Benjamini-Hochberg false-discovery rate below 5%.

## Results

### Generation of midbrain organoids

**Fig 1:**
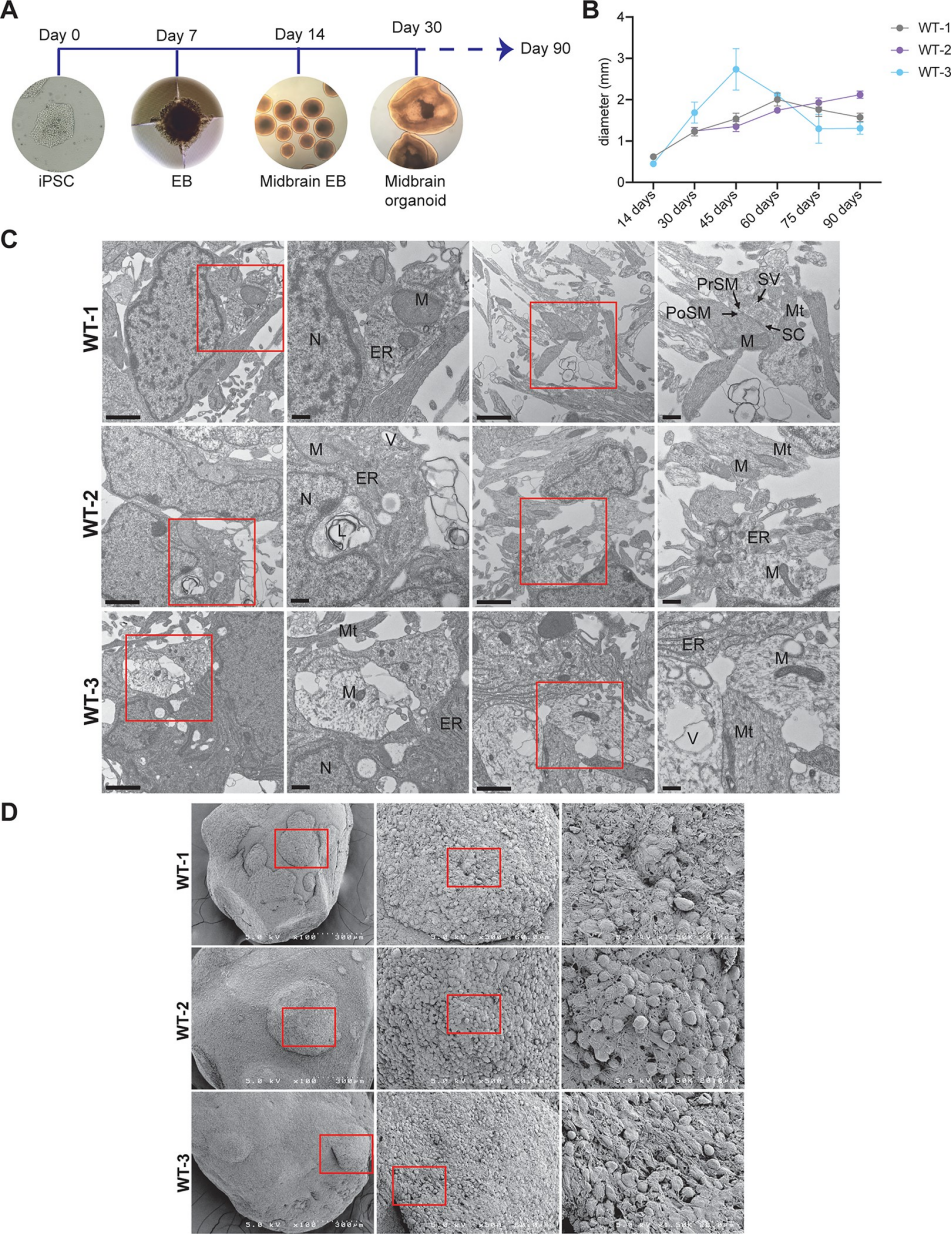
Midbrain organoid development. (A) A diagram illustrating the stages of MO development. (B) A graph tracking the change in MO size over time, presented as diameter (mm). (C) TEM images showing that MO cells contain major cellular features across all organoid lines (N—nucleus, ER—endoplasmic reticulum, M—mitochondria, V—vesicle,L—lysosome, Mt—microtubule, SC—synaptic cleft, PoSM—post-synaptic membrane, PrSM—pre-synaptic membrane, SV—synaptic vesicles). Scale bars: 2 μm (1000x), 500 nm (6000x). Red boxes indicate areas of magnification. (D) SEM images show MOs have similar phenotypes, with a healthy network of neuronal cells characterized by large protrusions and fibrous connection between cells. Red boxes indicate areas of magnification.

MOs were generated by following the protocol described in Qian et al [20]. Briefly, hiPSC were cultured for 7 days to generate embryoid bodies (EBs). Matrigel embedded EBs were then allowed to form midbrain-specific EBs, dissociated and transferred to a spin bioreactor to form midbrain-specific organoids which were grown for up to 90 days (Fig 1A). The midbrain organoids grew to 2–3 mm in diameter that reached their peak size in 60 days (Fig 1B). To further characterize the development of midbrain organoids, we performed immunohistochemical analysis for different protein markers (TH, FOXA2, GABA, GFAP, tubulin and caspase) at different time points. The expression profile of these proteins is discussed in detail in this article.

### Electron microscopy reveals neuronal characteristics of midbrain organoids

The intracellular and extracellular phenotypes of mature 90-day MOs were evaluated by transmission electron microscopy (TEM) and scanning electron microscopy (SEM) respectively. Common cellular features such as nuclei, endoplasmic reticulum, mitochondria, lysosomes and vesicles can be found throughout the cells of MOs (Fig 1C). Additionally, specific neuronal features such as microtubules and fully developed synaptic clefts can also be found (Fig 1C). The surface of each MO line was characterized by healthy well-defined cell bodies that are surrounded by a dense network of processes-like protrusions. Phenotypes were consistent across the three wildtype lines for both SEM and TEM and results mirror those that have been observed in neurons maintained in 2D cultures and those obtained from brain tissue [25, 26].

### Evaluation of overall midbrain organoid development

**Fig 2:**
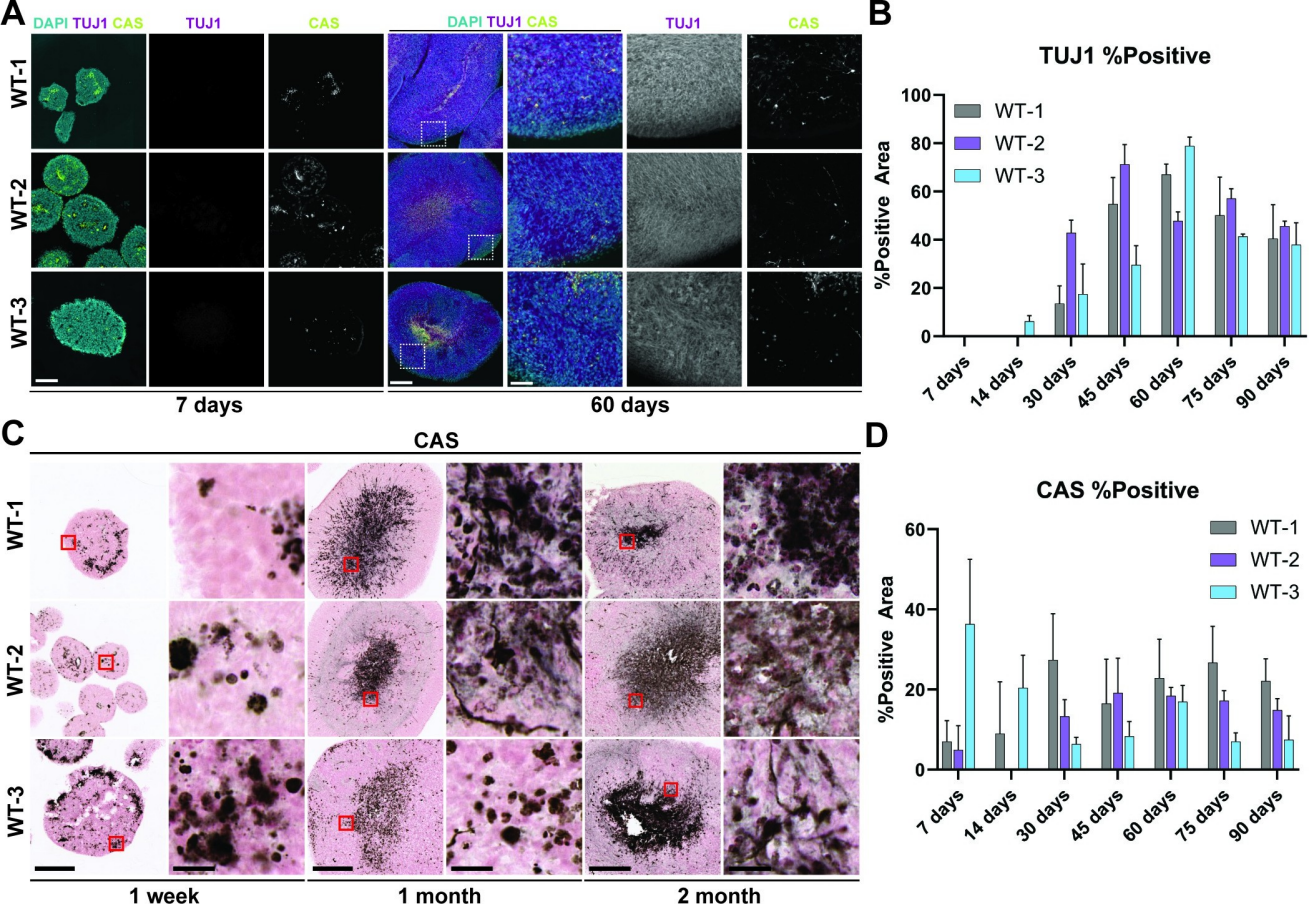
**Phenotype of MOs as determined by βIII Tubulin (TUJ1) and Caspase3 (CAS) immunofluorescence and immunohistochemistry.** (A) Immunofluorescence images TUJ1 (magenta) and CAS (yellow) staining in MOs of each line at 5x and 20x magnification, nuclei are stained with DAPI (cyan). Scale bars: 125 μm (5x), 25 μm (20x). Regions of magnification are indicated with white dashed boxes. (B) Total TUJ1-positive area for every line at every time point, data is presented as a bar plot of the mean ± s.d. of the percentage of the total tissue area that was stained positive for TUJ1 for each line. (C) Immunohistochemistry of CAS staining in each organoid line at 4x and 40x magnification. Scale bars: 250 μm (4x), 25 μm (40x). Regions of magnification are indicated with solid red boxes. (D) Total CAS-positive area with data presented as a bar plot of the mean ± s.d. of the percentage of the total tissue area that was stained positive for CAS for each line.

To assess the development and health of the WT organoid they were co-stained with βIII Tubulin (TUJ1), and cleaved Caspase 3 (CAS) (Fig 2A). TUJ1 is a neuron-specific isoform of tubulin that is especially prevalent in neurons that are newly generated during fetal brain development or from adult neuron regeneration [27]. The development of robust TUJ1 staining by 30 days confirmed the maturation of the EBs into MOs and the generation of neurons in all WT line organoids (Fig 2B). WT-2 shows the largest induction in TUJ1 expression relative to the other lines (Fig 2B), but all three lines show comparable levels of TUJ1 staining at 60 days. Additionally, the distribution of TUJ1 staining was not different across the organoid lines with dense staining visible throughout the organoids of each line (Fig 2A).

CAS activation is a marker of apoptosis and was used to determine the overall health of the organoids throughout their maturation. CAS staining remains sparse throughout the organoids of each line until around 30 days when a densely stained region forms at the center of most organoids in each line (Fig 2C). However, all lines express a relatively stable amount of CAS over the 90 days of development ranging from 10–20%, with two exceptions at 1 week for WT-3 and at 30 days for WT-1 (Fig 2D). This indicates that the majority of the cells in the organoids remain healthy through 90-days of differentiation despite the increasing size of the organoid.

### Midbrain organoids develop dopaminergic neurons

**Fig 3:**
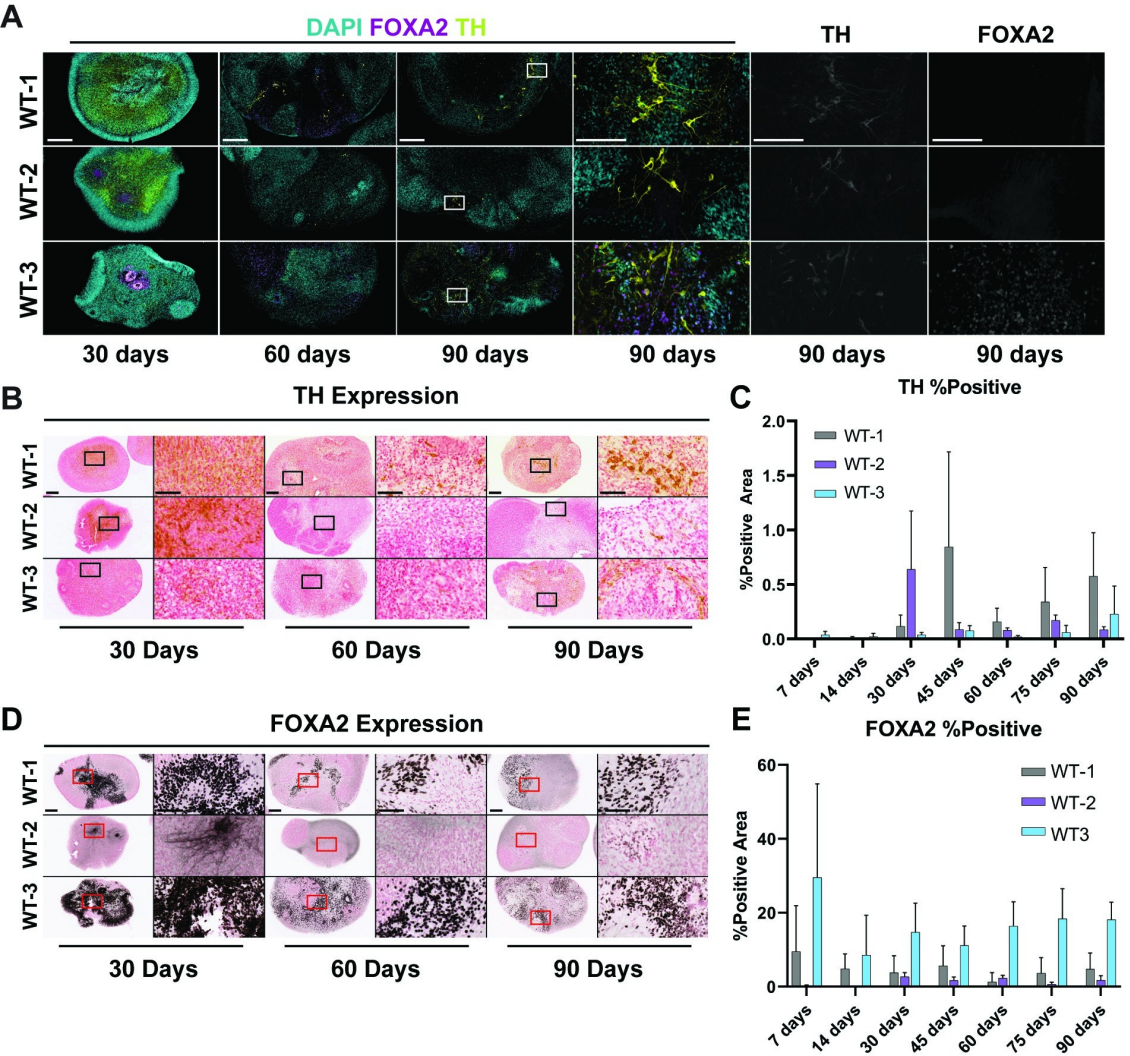
Development of TH and FOXA2 in MOs. (A) Immunofluorescence images of FOXA2 (magenta) and TH (yellow) staining in MOs of each line at 5x and 20x magnification, nuclei are stained with DAPI (cyan). Regions of magnification are indicated with white dashed boxes. Scale bars: 250 μm (5x), 50 μm (20x). (B) Brightfield images of TH immunohistochemistry staining at 4x and 20x. Scale bars: 125 μm (5x), 25 μm (20x). Regions of magnification are indicated with solid black boxes. Scale bars: 250 μm (4x) and 50 μm (20x). (C) Total TH-positive area with data presented as a bar plot of the mean ± s.d. of the percentage of the total tissue area that was stained positive for TH for each line. (D) Brightfield immunohistochemistry staining of FOXA2 at 4x and 20x. Scale bars: 250 μm (5x), 50 μm (20x). Regions of magnification are indicated with solid red boxes. (E) Total FOXA2-positive area for every line at every time point, data is presented as a bar plot of the mean ± s.d. of the percentage of the total tissue area that was stained positive for FOXA2 for each line.

The substantia nigra and the ventral tegmental area of the midbrain region express dopaminergic neurons that are critical for voluntary movements [28]. Forkhead box protein A2 (FOXA2) is a transcription factor that has an array of important roles in development and is a well characterized marker for dopaminergic neurons, along with tyrosine hydroxylase (TH), a key enzyme in the catecholaminergic pathway [29, 30]. TH expression levels are reported to be regulated by FOXA2 expression. To quantify dopaminergic neuron formation in midbrain organoids we performed immunohistochemical and immunofluorescent staining of dopaminergic neuron markers FOXA2 and TH (Fig 3A, 3B and 3D).

Our results indicate that all midbrain organoids develop dopaminergic neurons. TH expression in midbrain organoids was variable over the course of development. At 30 days WT-2 exhibited greater TH expression than WT-1 and WT-3. However, the trend changed between 45 days and 90 days with WT-1 showing the greatest amount of TH expression followed by a decrease in TH expression in WT-2 and WT-3. Between 60 days and 90 days WT-2 and WT-3 showed roughly equal levels of TH expression, while TH expression in WT-1 increased over these time points (Fig 3C). While the amount of TH positive neurons was variable, no difference in localization of TH signal was observed. These results indicate that all midbrain organoids develop dopaminergic neurons.

Characterization of FOXA2 expression indicated a loss of neural stem cells as midbrain organoids matured as seen by an overall decrease in FOXA2 expression in WT-1 and WT-3 from 7 days to 90 days (Fig 3E). No trend was observed with WT-2 as FOXA2 expression remained relatively consistent and below 5% over the entire course of development. Comparison of FOXA2 expression and TH expression for WT-1 and WT-3 are consistent with reports that FOXA2 and TH expression have an inverse relationship. As FOXA2 expression decreases we observe an increase in TH over the course of midbrain development (Fig 3C and 3E).

### Midbrain organoids develop GABAergic neurons

**Fig 4:**
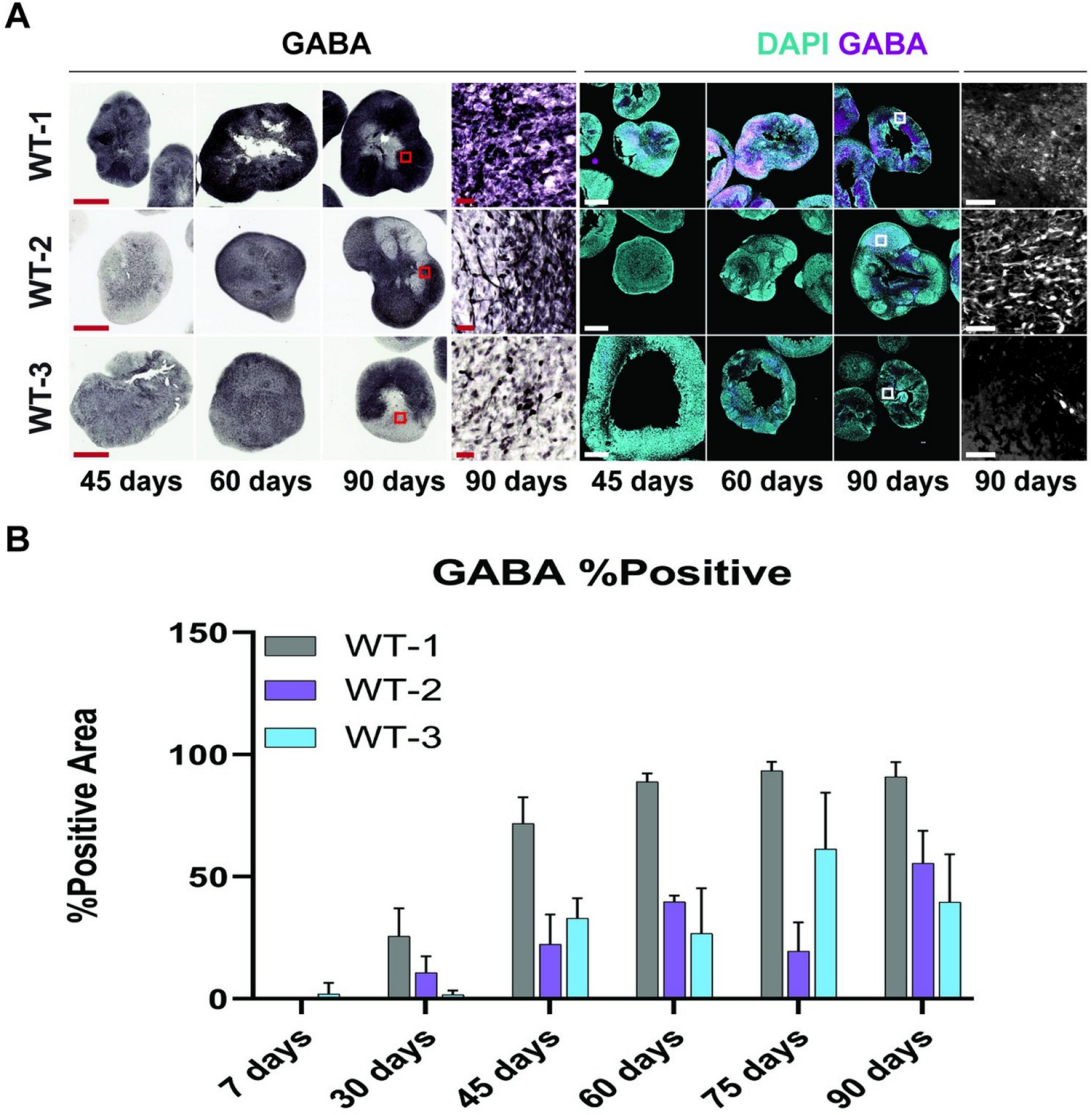
MOs contain GABAergic neurons. (A) Brightfield and immunofluorescence images for MOs stained for GABA (magenta) at different timepoints (45, 60 and 90 days), nuclei are stained with DAPI (cyan). Scale bars: 500 μm (for 45, 60 and 90 days) and 50 μm (for 90 days zoomed in image). Regions of magnification are indicated with solid red boxes. (B) Total GABA-positive area for every line at every time point, data is presented as a bar plot of the mean ± s.d. of the percentage of the total tissue area that was stained positive for GABA for each line.

GABAergic neurons are widely expressed in the midbrain region of the brain [31–33]. To characterize midbrain organoid development, we tracked the development of GABAergic neurons in the midbrain organoids (Fig 4). We performed immunohistochemistry for GABA at different time points and found that the midbrain organoids started showing significant expression of GABAergic neurons in 45 days old organoids and onwards and peaked at day 60 (Fig 4B). Although organoids generated from different iPSC lines showed varied levels of GABAergic neuron expression, the general trend in the expression pattern was similar through the development of midbrains at different timepoints.

### Astrocyte development in midbrain organoids

**Fig 5:**
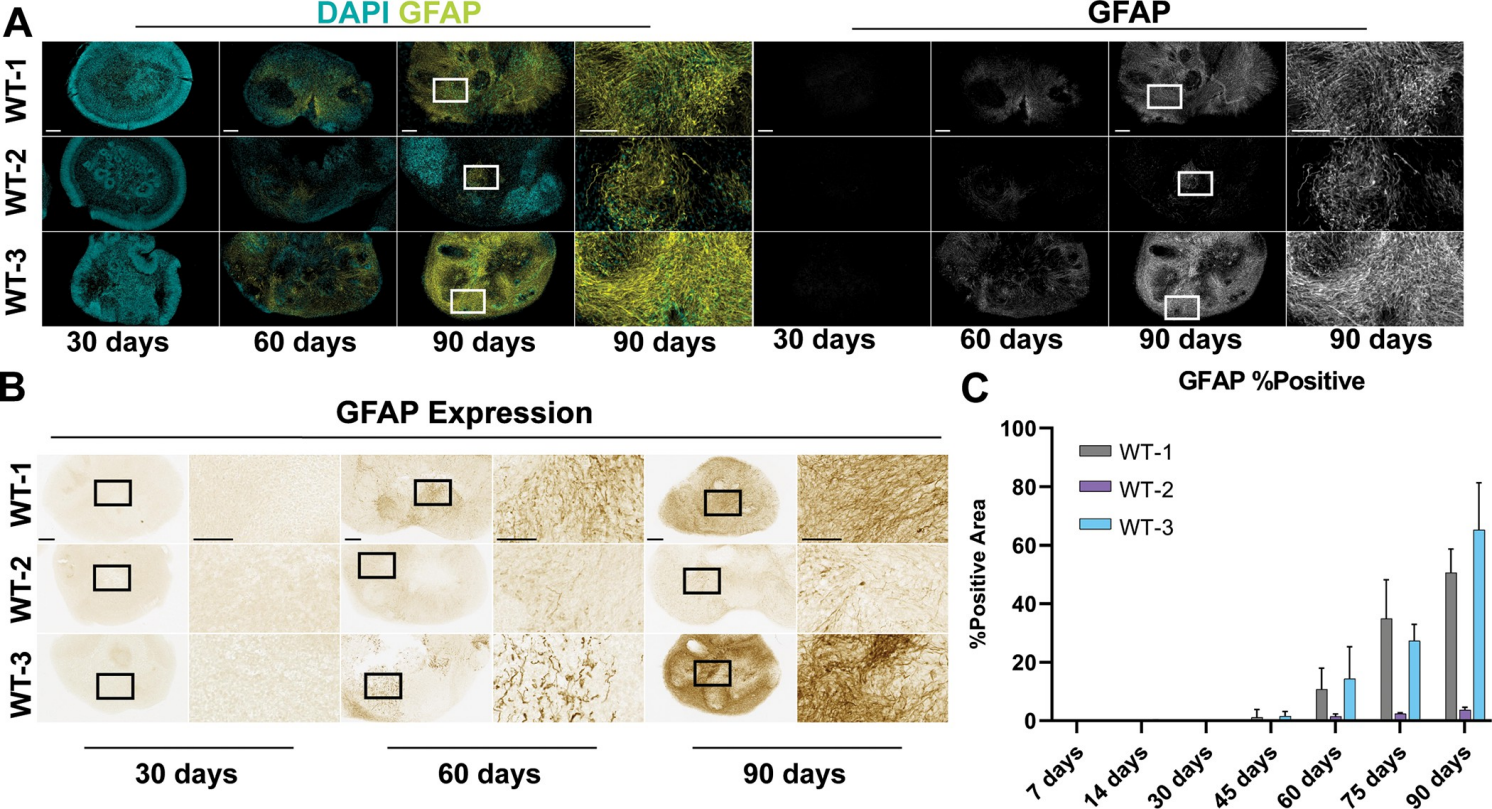
MOs develop astrocytes as shown by GFAP expression. (A) Immunofluorescence images for MOs stained with GFAP (yellow) at 5x and 20x magnification, nuclei are stained with DAPI (cyan). Scale bars: 250 μm (5x), 50 μm (20x). Regions of magnification are indicated with solid white boxes. (B) Brightfield immunohistochemistry images of GFAP staining at 4x and 20x. Scale bars: 250 μm (5x), 50 μm (20x). Regions of magnification are shown as solid black boxes. (C) Total GFAP-positive area for every line at every time point, data is presented as a bar plot of the mean ± s.d. of the percentage of the total tissue area that was stained positive for GFAP for each line.

Astrocytes are the majorly expressed cell type in the brain and are critical for brain function [34]. Since Glial fibrillary acid protein (GFAP) is an established marker for astrocytes, we performed immunofluorescent and immunohistochemical staining of GFAP to assess astrocyte development in our midbrain organoids (Fig 5A and 5B). GFAP expression was not observed until 45 days of growth, with only minor expression observed in WT-1 and WT-3 (Fig 5C). From 60 days to 90 days all midbrain organoids expressed GFAP. At 60 and 75-day time points, WT-1 had the greatest expression of GFAP followed by WT-3, while WT-2 expressed GFAP but it did not increase between 60 and 90 days. WT-3 exceeded WT-1 GFAP expression at 90 days. These results suggest that all midbrain organoids develop astrocytes, with WT-1 and WT-3 exhibiting the greatest number of astrocytes.

### Midbrain organoids express μ-opioid receptor (MOR)/

**Fig 6:**
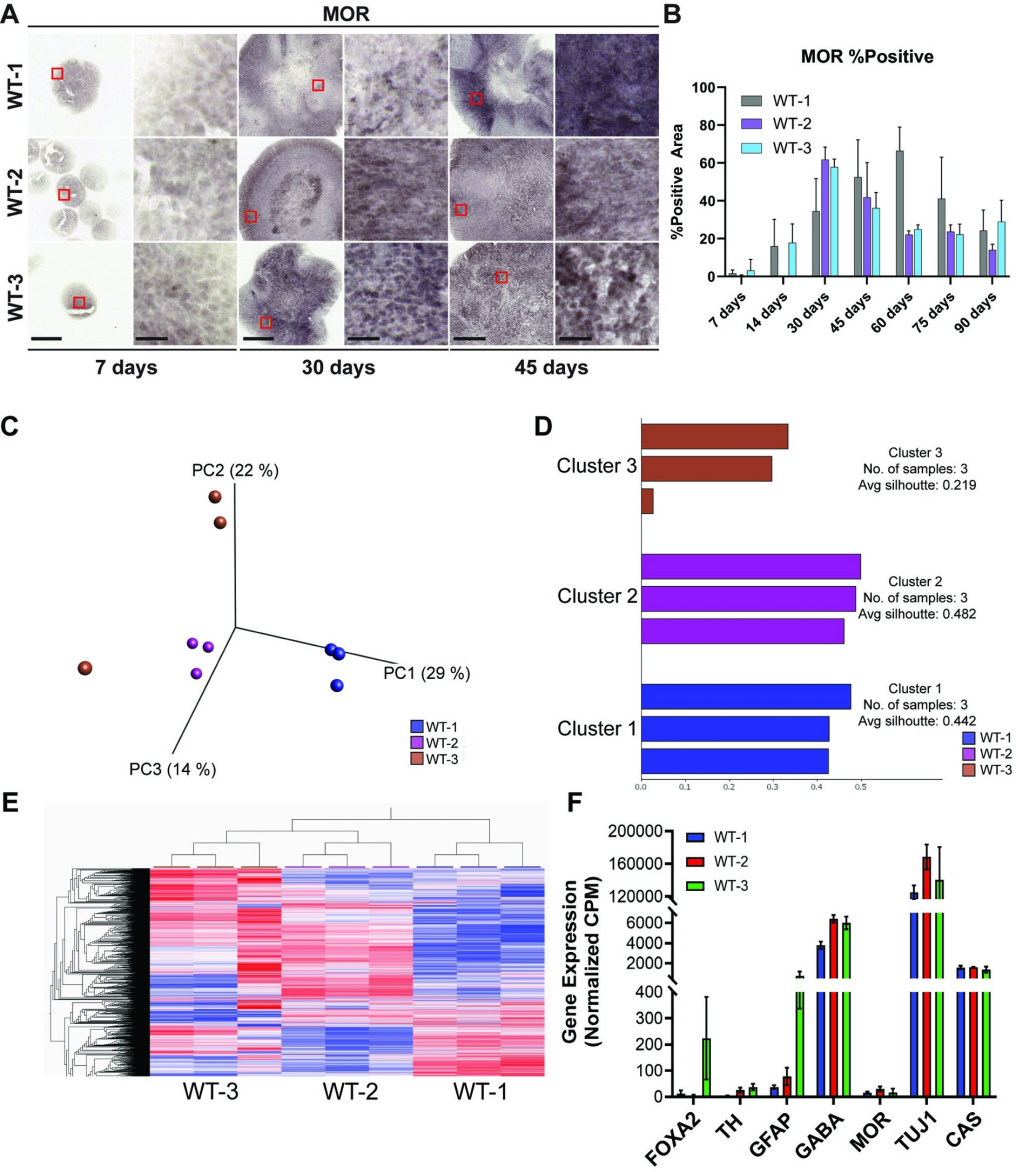
Development of MOR expression and global gene expression profiling at 90 days. (A) Immunohistochemistry of MOR staining in MOs at 4x and 40x magnification. Scale bars: 250 μm (4x), 25 μm (40x). Red boxes indicate regions of magnification. (B) Total MOR-positive area for every line at every time point, data is presented as a bar plot of the mean ± s.d. of the percentage of the total tissue area that was stained positive for MOR for each line. (C) Principal component analysis of midbrain organoids gene expression profiles for WT-1, WT-2, and WT-3. (D) Silhouette clustering analysis of k-means clustering of gene expression profiles of midbrain organoids WT-1, WT-3, and WT-3. (E) Hierarchical clustering of global gene expression profiles of midbrain organoids WT-1, WT-2, WT-3. (F) Normalized gene expression measured by counts per million (CPM) of FOXA2, TH, GFAP, GABA, MOR, TUJ1, and CAS.

MOR is central to the pain and reward circuits of the brain [35]. The activation of MOR is strongly agonist-dependent and endogenous ligand binding leads to the rapid internalization and inactivation of MORs. This regulatory mechanism is absent or greatly reduced for alkaloid opiates, such as morphine, and thus MOR is also involved in opioid addiction [36]. Previous studies have established MOR regulation of dopaminergic and GABAergic neurons, and its expression in the midbrain region of rats, thus the WT organoids were stained for the presence of MOR [36, 37]. Though all WT lines develop some MOR expression its development and total quantity is heterogeneous across the lines (Fig 6A and 6B). WT-1 seems to develop the largest quantity of MOR expression by 45 days (Fig 6B). It is also important to note, that while in WT-1 and WT-2, MOR seems to localize to specific regions of the organoid, similar to other neuron specific stains, this is not observed for WT-3 (Fig 6A). This heterogeneity may be important for understanding the genetic component (genetic background in each person) underlying opioid addiction. It also remains to be determined which time point best recapitulates the in vivo phenotype of the human brain, as MOR expression diminishes as the organoids grow more mature.

### RNA-seq profiling of 90-day midbrain organoids

RNA-seq analysis was performed on midbrain organoids harvested at 90 days (Fig 6C–6F). Principal component analysis (Fig 6C), k-means clustering (Fig 6D), and hierarchical clustering (Fig 6E) identified three distinct molecular phenotypes for the three midbrain organoid lines indicating that genetic variation of the initial iPSCs is maintained throughout midbrain organoid development. This data also demonstrates the reproducibility of the differentiation protocol, as almost all replicate RNAseq profiles closely match those derived from the same iPSC line as shown by k-means clustering. Lastly, RNA-seq analysis of FOXA2, TH, GFAP, GABA (GABBR1), MOR (OPRM1), TUJ1 (TUBA1A), CAS3 (CASP3) are consistent with the relative expression levels observed using imaging-based methods (Fig 6F).

## Discussion

Despite being less than a decade old, iPSC-derived brain organoids are quickly becoming a valuable tool for disease modeling and drug discovery. This is largely due to their ability to reliably express mature neuronal markers and recapitulate human-like brain structure and function in comparison to 2D cell culture and animal models. Initially, brain organoids were generated using mostly undirected methods, but recent innovations have allowed for the production of regiospecific brain organoids, such as midbrain organoids, that allow for the evaluation of therapeutics or the effects of a disease on a more focused population of neuronal cells. In this paper, we showed how midbrain-specific organoids can be made from several genetically distinct iPSC lines in order to provide a benchmark for a comparison of organoids generated from other protocols and cell lines can be evaluated.

Overall, this protocol was able to produce uniform, healthy organoids between different batches of the same iPSC-line and between different lines as measured by their total size, TEM, SEM and immunostaining. Our TEM data showed that for all three lines we observed all common intracellular cellular features in addition to neuron-specific synaptic clefts and microtubules. Across the three lines the SEM images showed that the organoids were composed of round healthy cells surrounded by dense networks of processes. Additionally, TUJ1 and CAS staining showed that at two months MOs from all three lines were composed of a majority of healthy, neuronal cells. RNAseq data further confirms the robustness of this protocol as all batches of the same line cluster together as determined by k-means clustering, except for a single batch of WT-3 MOs.

The three lines displayed higher variability for more mature neuronal markers and certain markers reach a peak in expression earlier than the 90-day timepoint. While this protocol was successful in inducing the expression of GABA, GFAP and MOR, the overall low level of TH positivity (<1%) does indicate that it is not optimal for the production of dopaminergic neurons compared to alternative protocols [15–18]. However, this lower enrichment may more closely represent the physiological level of dopaminergic neurons found in the brain where the highest proportion is found in the substantia nigra and does not exceed more than 5% of the total population [38]. After 30 days, WT-1 outperformed the other two WT MOs for the expression of TH, GABA and MOR. The decline in MOR and TH expression at the later time points can be partially explained for WT-1 and WT-3 by the increase in GFAP staining, as this indicates that later stage organoids are enriched in astrocytes, which do not express mature neuronal markers. A more mixed population of cells that include neurons and glia may be desirable as it better represents in vivo conditions where more than half of all cells in the brain are non-neuronal cells [39–41]. Inclusion of additional glial stains would be useful in future studies to determine if this protocol can also induce the formation of other glial cells such as microglia.

Although the three lines we used for this protocol were derived from fibroblasts from apparently healthy individuals, underlying genetic variation can still impact the ability of these lines to express certain markers and drive phenotypic differences, as shown by the RNAseq analysis. Additionally, it is well known that iPSC lines from the same donor can also show differences in differentiation efficiency due to SNVs acquired during reprogramming. Still, our study validates the ability of these three iPSC lines to differentiate into MOs and these lines can hopefully be used by the broader community as healthy controls for the study of neurological diseases. Particularly the RNAseq data set can be included in future bioinformatics studies as additional controls in order to further increase the power of any analyses.

However, a limitation of our current study is our reliance on a single protocol for MO generation. We cannot conclude that an alternative protocol would not yield better results for these iPSC lines. The protocol presented in this manuscript uses bioreactors to enable matrix free organoid generation, and thus it requires additional materials and skills compared to alternative methods. Therefore, it is important that direct comparisons are undertaken in the future to determine if the extra effort improves the overall physiological characteristics of the generated brain organoids, and we hope that the data presented here can be useful for these studies. We also focused on characterizing 2D slices of our MOs, which may not give an accurate representation of the overall distribution of different cell types. Therefore, it is also important to adapt clearing and imaging protocols that will allow for full 3D reconstruction of immunostaining [19]. Additionally, any future effort using this protocol should also include functional readouts such measurement of electrophysiology, and dopamine and neuromelanin production as presented in other manuscripts describing MO generation [15–19].

## Supporting information

S1 Data (CSV)
